# Activity-Dependent Pre-miR-134 Dendritic Localization Is Required for Hippocampal Neuron Dendritogenesis

**DOI:** 10.3389/fnmol.2018.00171

**Published:** 2018-06-11

**Authors:** Federico Zampa, Silvia Bicker, Gerhard Schratt

**Affiliations:** Institute of Physiological Chemistry, Philipps-University Marburg, Marburg, Germany

**Keywords:** dendritogenesis, microRNA, neuronal activity, BDNF, NMDA receptor, RNA transport, neuronal development

## Abstract

microRNAs (miRNAs) have emerged as critical regulators of neuronal dendrite development. Specific precursor (pre-)miRNAs are actively transported to dendrites, but whether this process is regulated by neuronal activity and involved in activity-dependent dendritogenesis is unknown. Here we show that BDNF, a neurotrophin that is released in response to increased neuronal activity, promotes dendritic accumulation of pre-miR-134. Dendritic accumulation, but not transcription of pre-miR-134, is abrogated by treatment of neurons with the NMDA receptor (NMDAR) antagonist APV. Furthermore, APV interferes with BDNF-mediated repression of the known miR-134 target Pumilio 2 (Pum2) in a miR-134 binding site-specific manner. At the functional level, both APV treatment and knockdown of the pre-miR-134 transport protein DHX36 antagonize BDNF-induced dendritogenesis. These effects are likely mediated by reduced dendritic miR-134 activity, since both transfection of a synthetic miR-134 duplex or of a dendritically targeted pre-miR-134-181a chimera rescues BDNF-dependent dendritogenesis in the presence of APV. In conclusion, we have identified a novel NMDAR-dependent mechanism involved in the activity-dependent control of miRNA function during neuronal development.

## Introduction

Neuronal dendrites are branched extensions of neurons that receive electrochemical stimulation from neighboring neurons via synaptic connections that mostly form on tiny protrusions known as dendritic spines. Extension and branching of dendrites, collectively referred to as dendritogenesis, is tightly regulated during neuronal development by both intrinsic mechanisms and external cues. In particular, sensory experience controls dendritogenesis during critical periods of development to shape functional neural circuits in response to environmental stimuli (Koleske, 2013; Ledda and Paratcha, 2017). Deficits in activity-dependent dendritogenesis are common hallmarks of many neurodevelopmental and neurodegenerative disorders (Kulkarni and Firestein, 2012; Martínez-Cerdeño, 2017). microRNAs (miRNAs) are a large class of small non-coding RNAs that inhibit the expression of specific target genes by recruiting the miRNA—induced silencing complex (miRISC) to the 3′ untranslated region (3′UTR) of the respective mRNA. Several hundred miRNA genes are expressed in post-mitotic neurons, and a specific subgroup is enriched in dendrites, where they are involved in dendritogenesis, spine development and synaptic plasticity (McNeill and Van Vactor, 2012). A well-studied example is miR-134, which is regulated by neuronal activity and required for activity-dependent dendrite growth, dendritic spine development and homeostatic synaptic downscaling (Schratt et al., 2006; Fiore et al., 2009, 2014). Accordingly, inhibition of miR-134 *in vivo* attenuated seizure activity in a temporal lobe epilepsy model and restored contextual fear memory formation in SIRT1-deficient mice (Gao et al., 2010; Jimenez-Mateos et al., 2012). Several critical miR-134 target genes have been identified, including the RNA-binding protein (RBP) Pumilio-2 (Pum2), Limk1 and Creb1.

Mature miRNAs result from a biogenesis program that involves two sequential cleavage steps: the nuclear “cropping” of the primary transcript originating from the miRNA gene (the pri-miRNA) into a pre-miRNA, and the cytoplasmic “dicing” of the latter into a miRNA duplex, one strand of which is subsequently loaded into miRISC (Kim, 2005; Ha and Kim, 2014). Several studies identified pre-miRNAs along with protein components of the pre-miRNA processing complex in neuronal dendrites (Lugli et al., 2008; Bicker et al., 2013; Sambandan et al., 2017). Therefore, it was hypothesized that a specific subset of pre-miRNAs could undergo localized processing in dendrites, which in turn could create an additional layer of local protein synthesis regulation in response to neuronal activity. Data supporting this hypothesis was recently obtained for pre-miR-181a, which was shown to undergo activity-dependent processing in dendrites upon mono-synaptic stimulation followed by increased inhibition of its target mRNA CamK2α (Sambandan et al., 2017).

In contrast to processing, little is known about the mechanism of pre-miRNA localization to dendrites. We recently showed that dendritic accumulation of pre-miR-134 requires binding of the RNA helicase DHX36 to a specific sequence motif within the pre-miR-134 terminal loop (Bicker et al., 2013). However, whether dendritic localization of pre-miRNAs is regulated by neuronal activity, as well as potential functional implications of such a regulation, are unknown.

In this study, we tested the hypothesis that pre-miR-134 transport to dendrites is regulated by neuronal activity. Therefore, we focused on the neurotrophin BDNF, which is synthetized and released from neurons in response to neuronal activity and whose expression is needed for survival and induction of dendritic growth in immature neurons (Park and Poo, 2013). Our previous results further suggest that BDNF is implicated in the regulation of miR-134 activity. First, BDNF treatment of primary cortical neurons induces transcription of the miR-379-410 cluster, which contains the miR-134 gene. Second, BDNF induces dendritogenesis by repressing Pum2 in a miR-134-dependent manner (Fiore et al., 2009). Third, BDNF attenuates silencing activity of a miR-134-containing miRISC on the dendritic target mRNA Limk1 (Schratt et al., 2006).

We therefore considered that BDNF-dependent modulation of the pre-miR-134 dendritic supply could provide a mechanism to adjust the repressive activity of miR-134 during activity-dependent neuronal development.

## Materials and Methods

### Primary Rat and Mouse Neuronal Cultures

Euthanasia of pregnant rats and newborn mice for brain tissue preparation was approved by the animal committee at the Regierungspräsidium Giessen.

Primary hippocampal and cortical neuronal cultures were prepared from embryonic day 18 (E18) Sprague–Dawley rats (Charles River Laboratories) and plated in 24 multi-well plates as described (Schratt et al., 2006). For neuronal cultures on compartmentalized chambers, cells were plated on polyethylene terephthalate (PET) membrane cell culture inserts (pore size: 1 μm, diameter: 23 mm (Corning)) that were matrix-coated as previously described (Bicker et al., 2013).

Primary cultures of mouse hippocampal neurons were prepared from P1 pups according to the rat protocol, but with the following adaptations: each dissected hippocampus was collected in Leibovitz’s L15 medium (Life Technologies) with 7 mM HEPES. After medium removal, 500 μl of TrypLE Express were added for 7 min at 37°C and mixed by inversion for three times every minute. After mechanical dissociation, mouse hippocampal neurons were plated as described for rat neurons.

### Fluorescence *in Situ* Hybridization (FISH)

Day *in vitro* (DIV) 7 rat hippocampal neurons were treated with 50 ng/ml recombinant human BDNF (PeproTech) and fixed after 2 h. Fluorescence *in situ* hybridization (FISH) was performed as previously described (Bicker et al., 2013), using 5’FITC-labeled LNA probes and a 2-step antibody amplification for detection.

### Single-Molecule Fluorescence *in Situ* Hybridization (smFISH)

DIV6 mouse hippocampal neurons were treated with 100 ng/ml BDNF for 2 h. Single-molecule fluorescence *in situ* hybridization (smFISH) was performed using the QuantiGene ViewRNA ISH Cell Assay kit (Affymetrix) as previously described (Valluy et al., 2015), but omitting the protease step to preserve dendrite integrity. The Exon134_485 probe (Type 4, Alexa488) was designed to target 836 bases spanning the miR-134- and miR-485-containing exons of the Mirg transcript.

### Drug Treatment of Compartmentalized Chambers

DIV19 hippocampal neurons grown in compartmentalized chambers were pre-treated with 50 μM DL-APV (Tocris) or Mock (water) for 30 min in both compartments and treated with 75 ng/ml BDNF or Mock (water) for 2 h either in the cell body or in the process compartment. RNA was separately extracted from the two compartments and processed for qPCR as previously described (Bicker et al., 2013).

### qPCR

RNA extraction was performed using peqGOLD TriFast (VWR) or mirVana miRNA isolation kit (Ambion) following manufacturers’ instructions. Reverse transcription and qPCR were performed and analyzed as described (Bicker et al., 2013). Gapdh (pre-miRNAs) and miR-124 (miRNAs) were used as reference genes for qPCRs performed in compartmentalized cultures, U6 for qPCR in whole cells. Oligonucleotide sequences are reported in the Supplementary Material.

### Cy3-Labeled Pre-miRNA Probes: Synthesis, Transfection and Drug Treatment

Cy3-pre-miR-134 and the respective chimeras were *in vitro* synthesized with the MEGAshortscript T7 kit (Ambion) from oligonucleotide templates and labeled with the Label IT nucleic acid labeling kit (Mirus) as previously described (Bicker et al., 2013). DIV7 rat hippocampal neurons were transfected with 75 nM Cy3-pre-miRNAs in NB+ without antibiotics using siPORT NeoFX (Ambion) as previously described (Bicker et al., 2013). Cells were pre-incubated in transfection medium containing 50 μM DL-APV or Mock for 30 min and then transfected and treated simultaneously with 50 ng/ml BDNF or Mock for 3 h. Cells were fixed in 4% paraformaldehyde/4% sucrose/DEPC-PBS and stained with mouse monoclonal α-MAP2 (1:2000, Sigma Aldrich, M9942) and rabbit polyclonal α-cFos (1:1000, Cell Signalling #2250) as previously described (Fiore et al., 2009).

### Plasmids

pSuper constructs and luciferase pGL4-PEST reporter plasmids have been previously validated and described (Bicker et al., 2013; Fiore et al., 2014). To create the pri-miR-134 expressing plasmid, a sequence spanning 150 bp on either side of the pre-miR-134 stem loop was amplified from the rat genome and cloned into pcDNA3 using HindIII/XhoI restriction sites. To create the pri-miR-134L150 and pri-miR-134L181a chimeras, overlapping primers were designed centered on the pre-miR-134 loop and used in combination with the primers used for pri-miR-134 amplification. The resulting product was cloned into pcDNA3. The PGK promoter of the pmirGLO vectors used in Figure 5E was substituted with a SV40 promoter (BglII/HindIII sites), to have both luciferase genes under the SV40 promoter. An oligonucleotide containing three consecutive miR-134 perfect binding sites (3x-miR-134-PBS) or the corresponding reverse sequence (3x-miR-134-REV) were inserted in the multiple cloning site using XbaI/SalI restriction sites. Oligonucleotide sequences are reported in the Supplementary Material.

**Figure 1:**
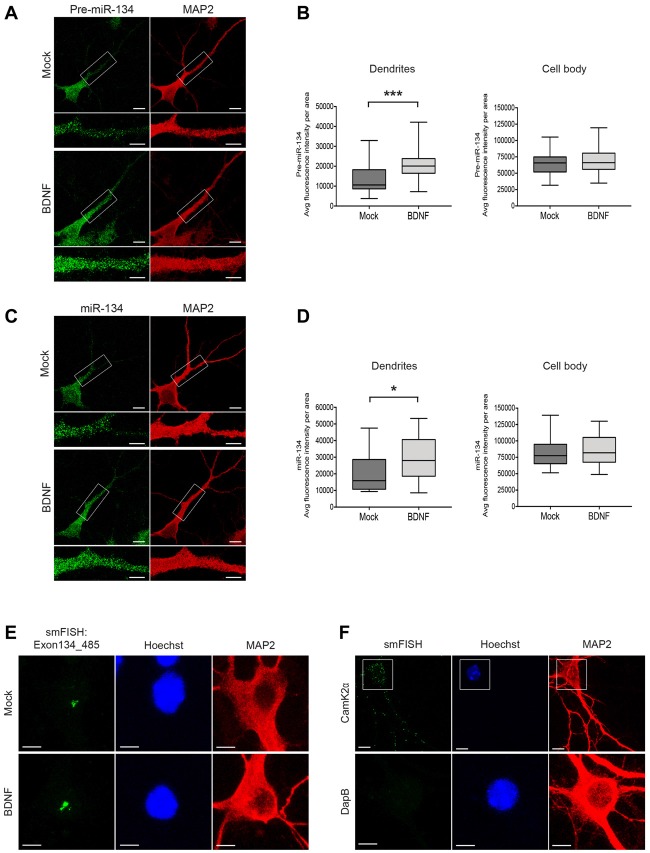
Short-time BDNF treatment induces pre-miR-134 dendritic accumulation. **(A,C)** Representative pictures from fluorescence *in situ* hybridization (FISH) using probes directed against the pre-miR-134 loop **(A)** or mature miR-134 **(C)** performed in developing rat hippocampal neurons (DIV7) treated with BDNF for 2 h (scale bar: 10 μm). Inserts at higher magnification illustrate the dendritic accumulation of pre-miR-134 granules. MAP2 staining (red) was used to visualize dendritic processes (scale bar: 5 μm). **(B,D)** Quantification of pre-miR-134 **(B)** or mature miR-134 **(D)** FISH signal in dendrites (left) or cell bodies (right) of multiple neurons indicated in **(A,C)**. **(B)** Mock *n* = 27, BDNF *n* = 27. **(D)** Mock *n* = 27, BDNF *n* = 28 cells analyzed from *n* = 3 independent biological replicates. Mann-Witney test, *P < 0.05, ***P < 0.001. **(E,F)** BDNF-induced increase in pri-miR-134 is confined to the nucleus. **(E)** Representative pictures of single molecule FISH (smFISH) with probes spanning Exon134_485 of the Mirg transcript. Pictures show a magnified region of the neuronal cell body, no signal was detected in dendrites (scale bar: 5 μm). **(F)** Representative pictures of smFISH with probes specific for Camk2α (positive control for dendritic RNA localization, scale bar: 10 μm; top) or DapB (bacterially expressed gene used as a negative control, scale bar: 5 μm; bottom).

**Figure 2:**
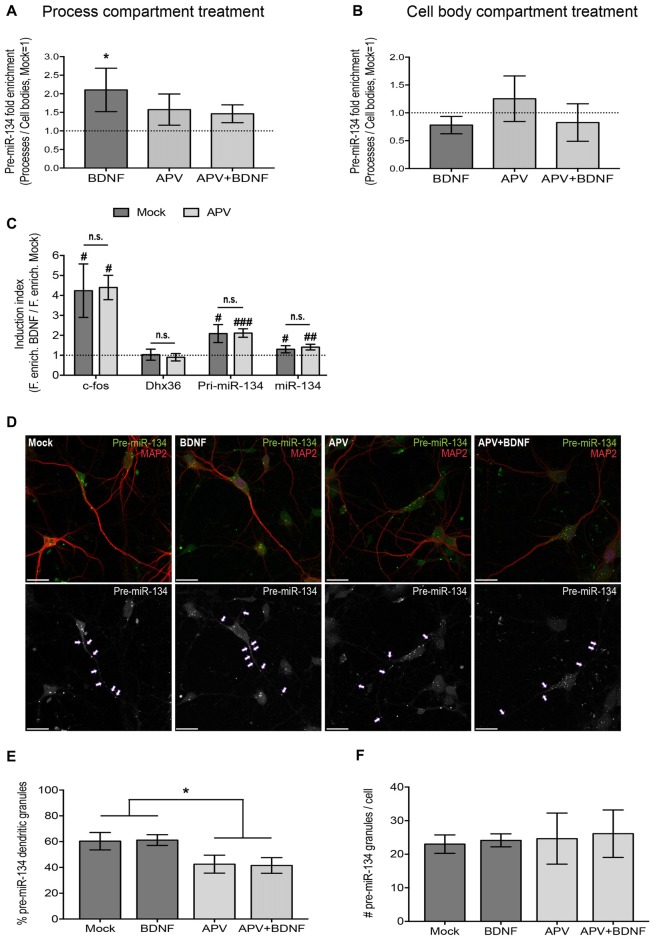
NMDA receptor (NMDAR) blockade impairs dendritic accumulation of pre-miR-134. **(A,B)** qPCR using RNA extracted from both the process and cell body compartment of DIV19 hippocampal neurons cultured on filter inserts and treated in both compartments with APV/Mock for 30 min and with BDNF/Mock for 2 h selectively in the process **(A)** or cell body **(B)** compartment. Values represent the mean of the relative process enrichment (ratio process vs. cell body expression) of pre-miR-134 ± SD. Values obtained for Mock-treated neurons are set to one. *n* = 3 independent experiments. One-way ANOVA and Tukey’s *post hoc* test, *P < 0.05. **(C)** qPCR for indicated genes using RNA extracted from DIV5 cortical neurons treated with BDNF (3 h) in the presence or absence (Mock) of APV. Values represent the mean BDNF induction index (ratio fold enrichment BDNF vs. Mock) ± SD of *n* = 4 independent biological replicates. Mock vs. APV: two-sample students *t*-test (n.s.). BDNF vs. Mock: one-sample or two-sample students *t*-test, ^#^*P* < 0.05, ^##^*P* < 0.01, ^###^*P* < 0.0001. **(D)** Representative pictures of DIV7 hippocampal neurons transfected with *in vitro* synthesized Cy3-pre-miR-134 and simultaneously treated with BDNF in the presence or absence of APV. Upper panel: Merged images of Cy3-pre-miR-134 (green), MAP2 (red). Lower panel: High contrast black-and-white images from the Cy3-pre-miR-134 channel only. Arrows point to dendritic pre-miR-134 granules (scale bar: 20 μm). **(E,F)** Quantification of multiple neurons shown in **(D)**. Values represent the percentage of dendritically localized **(E)** or the total number **(F)** of Cy3-pre-miR-134 granules per cell ± SD. *n* = 42 cells analyzed per data point from *n* = 3 independent biological replicates. One-way ANOVA and Tukey’s *post hoc* test, *P < 0.05.

**Figure 3:**
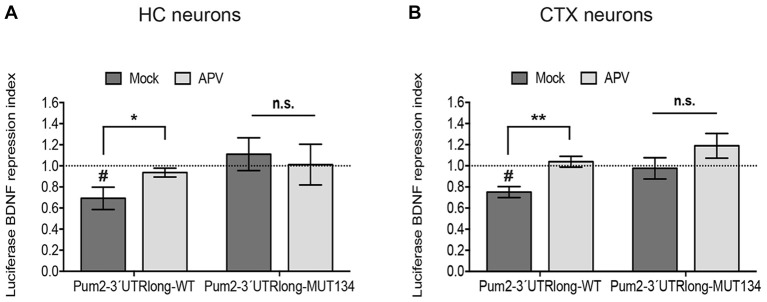
NMDAR blockade impairs miR-134-dependent repression of Pum2. Luciferase reporter gene assays performed in either DIV11 hippocampal neurons (HC) **(A)** or DIV6 cortical neurons (CTX) **(B)** treated for 6 h with BDNF in the presence or absence of APV. pGL4-PEST-Pum2-3′UTRlong firefly reporter (WT), pGL4-PEST-Pum2-3′UTRlong with mutated miR-134 binding site firefly reporter (MUT134) or pGL4-PEST empty vector were transfected together with a pGL4-Renilla reporter for internal normalization. Firefly/Renilla activity ratios were calculated for the Pum2 reporters and subsequently normalized to the respective ratio of pGL4-PEST empty. Values represent the mean BDNF repression index (ratio normalized luciferase activity BDNF vs. Mock-treated neurons) ± SD of *n* = 3 independent biological replicates. Induction indices: two-sample students *t*-test, *P < 0.05, **P < 0.01. BDNF vs. Mock: one-sample **(A)** or two-sample **(B)** students *t*-test, ^#^*P* < 0.05.

**Figure 4:**
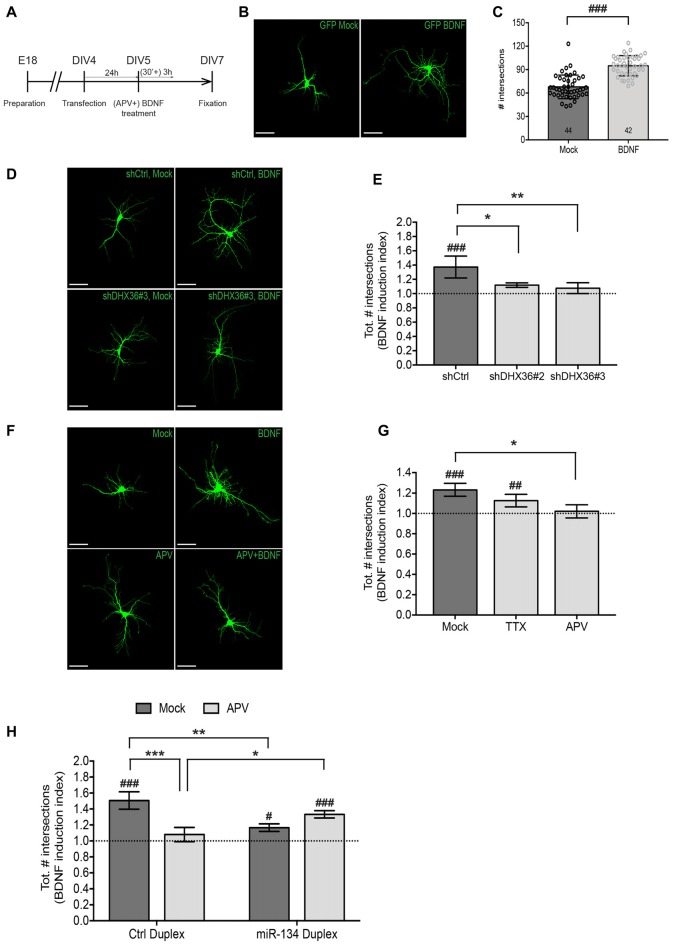
NMDAR-dependent dendritogenesis requires miR-134. **(A)** Experimental strategy to assess BDNF-induced dendritic outgrowth in immature neurons. Hippocampal neurons were transfected at DIV4, treated 24 h later for 3 h with BDNF in the presence or absence of APV (30 min pre-treatment) and fixed 48 h after the treatment. **(B)** Dendritic arborization of representative GFP-transfected hippocampal neurons (DIV7) processed as indicated in **(A)** without APV pre-treatment (scale bar: 50 μm). **(C)** Quantification of dendrite complexity of multiple neurons from **(B)** using Sholl analysis. Values represent the average total number of intersections obtained with Sholl analysis ± SD. *n* = 44 (Mock), *n* = 42 (BDNF) cells analyzed per data point from *n* = 4 independent biological replicates. Mann-Whitney test, ^###^*P* < 0.0001. **(D)** Dendritic arborization of representative GFP-transfected hippocampal neurons (DIV7) co-transfected with indicated shRNA constructs and further processed as indicated in **(A)** but without APV pre-treatment (scale bar: 50 μm). **(E)** Quantification of dendrite complexity of multiple neurons from **(D)** using Sholl analysis. Values represent average BDNF induction indices (total number of intersections in BDNF vs. Mock-treated neurons) ± SD of *n* = 4 independent biological replicates. Two-way ANOVA and Dunnet’s, *P < 0.05; **P < 0.01. BDNF vs. Mock: one-way ANOVA and Bonferroni, ^###^*P* < 0.001. **(F)** Dendritic arborization of representative GFP-transfected hippocampal neurons (DIV7) processed as indicated in **(A)** (scale bar: 50 μm). **(G)** Quantification of dendrite complexity of multiple neurons from **(F)** using Sholl analysis. Values represent average BDNF induction indices (total number of intersections in BDNF vs. Mock-treated neurons) ± SD of *n* = 3 independent biological replicates. Two-way ANOVA and Dunnet’s *P < 0.05. BDNF vs. Mock: one-way ANOVA and Bonferroni, ^##^*P* < 0.01, ^###^*P* < 0.001. **(H)** Quantification of dendrite complexity of neurons transfected with the indicated duplex RNA and treated with BDNF in the presence or absence of APV using Sholl analysis. Values represent the average BDNF induction indices (total number of intersections in BDNF vs. Mock-treated neurons) ± SD of *n* = 3 independent biological replicates. Number of analyzed cells and individual data points are indicated and analyzed in Supplementary Figure S3. Two-way ANOVA and Tukey’s, *P < 0.05, **P < 0.01, ***P < 0.001. BDNF vs. Mock: Kruskal-Wallis and Dunn’s, ^#^*P* < 0.05, ^##^*P* < 0.01, ^###^*P* < 0.001.

**Figure 5:**
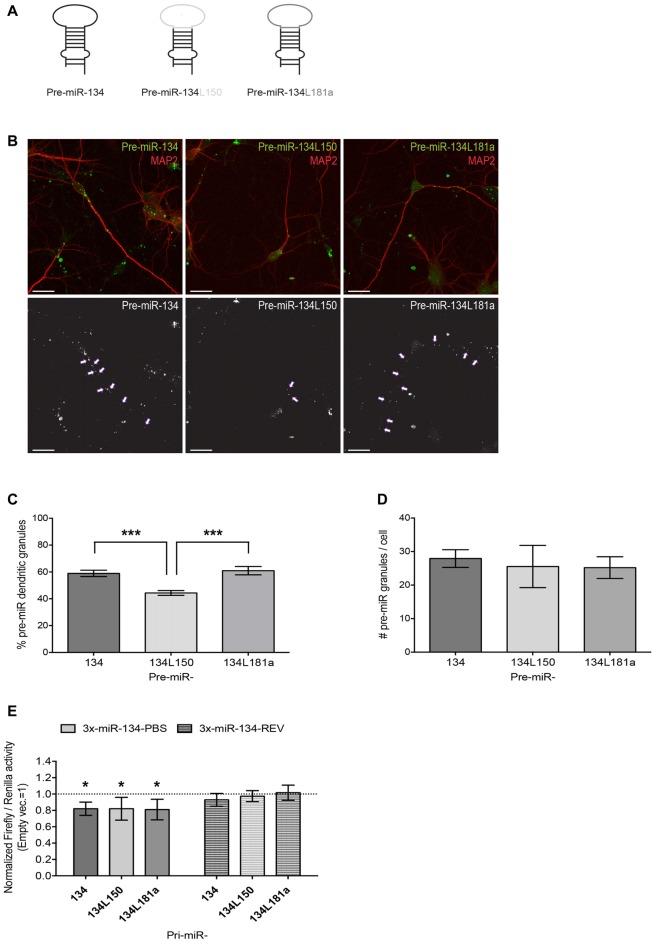
Design and validation of functionality and subcellular localization of pre-miR-134 chimeras. **(A)** Graphic representation of pre-miR-134 chimeras tested for subcellular localization. The pre-miR-134 terminal loop was substituted with either the one from pre-miR-150 (pre-miR-134L150) or the one from pre-miR-181a (pre-miR134L181a). **(B)** Representative pictures of developing hippocampal neurons transfected with *in vitro* synthesized Cy3-labeled pre-miRNAs described in **(A)**. Upper panel: merged images of Cy3-pre-miRNAs (green) and MAP2 antibody staining (red). Lower panel: high contrast black-and-white image of the Cy3-pre-miRNA signal only. Arrows point to dendritic pre-miR-134 granules (scale bar: 20 μm). **(C,D)** Quantification of dendritic pre-miRNA granules from multiple neurons shown in **(B)**. Values represent the average percentage of dendritically localized **(C)** or the total number/cell **(D)** of Cy3-pre-miRNA granules ± SD. *n* = 40 cells analyzed per data point from *n* = 3 independent biological replicates. One-way ANOVA and Tukey’s, ***P < 0.001. **(E)** Luciferase reporter gene assays performed using plasmids containing either 3xmiR-134 perfect binding sites (3x-miR-134-PBS) or the corresponding reverse sequence (3x-miR-134-REV) in the firefly luciferase 3′UTR. Firefly reporters were co-transfected with the indicated pre-miRNA expression vectors (or empty pcDNA3) and Renilla luciferase expression was used for internal normalization. Firefly/Renilla ratios were calculated for miR-134 reporters and subsequently normalized to the ratios obtained with the pmirGLO empty vector. The condition co-transfected with empty pcDNA3 was set to one (not shown). Values represent the mean normalized luciferase activity ± SD of *n* = 4 independent biological replicates. One-sample *t*-test, *P < 0.05.

### Dendritic Outgrowth Experiments: Transfections and Drug Treatments

DIV4 primary rat hippocampal neurons were transfected using Lipofectamine2000 (Invitrogen) as previously described (Valluy et al., 2015), with a transfection efficiency of about 5%. MiR-134 duplex (hsa-miR-134-5p Pre-miR miRNA Precursor, Ambion) and control duplex (Pre-miR Negative Control#1, Ambion) were transfected at a 2.5 nM final concentration. Twenty-four hours after transfection, cells were pre-treated with 50 μM DL-APV, 2 μM TTX (Tocris), or Mock for 30 min before treatment with 50 ng/ml BDNF or Mock for 3 h. After treatment, the medium was replaced with conditioned medium and cells were fixed 48 h later in 4% paraformaldehyde/4% sucrose/DEPC-PBS.

### Confocal Microscopy and Picture Analysis

#### FISH

For each data point, pictures of 8–10 pyramidal hippocampal neurons per experiment from three independent biological replicates were taken in a blinded manner with a LSM5 Pascal Zeiss confocal microscope using a 63× Oil objective at a resolution of 1024 (x/y) pixel, corresponding to an image size of 143 (x/y) μm. Pictures represent maximum projections of five consecutive optical sections taken at a 0.4 μm interval. Analysis of the FISH images was performed on z stack maximum projections. For quantification of the FISH signal, the average fluorescence intensity in dendrites and cell bodies was measured with the ImageJ software and normalized to the respective area using the MAP2 co-staining as mask. Number of analyzed cells per data point is indicated in the figure legend.

#### smFISH

Representative smFISH pictures were taken with a Leica SP5 confocal microscope equipped with a HyD hybrid detector using a 40× Oil objective at a resolution of 512 (x/y) pixel corresponding to an image size of 97 (x/y) μm. Pictures represent maximum projections of 15 consecutive optical sections taken at a 0.4 μm interval and enclosing the whole cells.

#### Subcellular Localization of Cy3-pre-miRs

For each data point, pictures of 12–14 developed pyramidal hippocampal neurons per experiment from three independent biological replicates were taken in a blinded manner on an LSM880 Zeiss confocal microscope (Figure 2D) and a LSM5 Pascal Zeiss confocal microscope (Figure 5B), using a 63× Oil objective with a resolution of 1024 (x/y) pixels, corresponding respectively to an image size of 135 (x/y) and 143 (x/y) μm. For BDNF- treated conditions, only c-Fos positive cells were analyzed. Pictures represent maximum projections of eight consecutive optical sections taken at 0.45 μm intervals and enclosing the whole cells. Analysis was performed on z stack maximum projections. Dendritic percentage of Cy3-pre-miR granules was assessed with the ImageJ “analyze particles” function as previously described (Bicker et al., 2013). Number of analyzed cells per data point is indicated in the figure legends.

#### BDNF-Induced Dendritic Outgrowth

**Figure 6:**
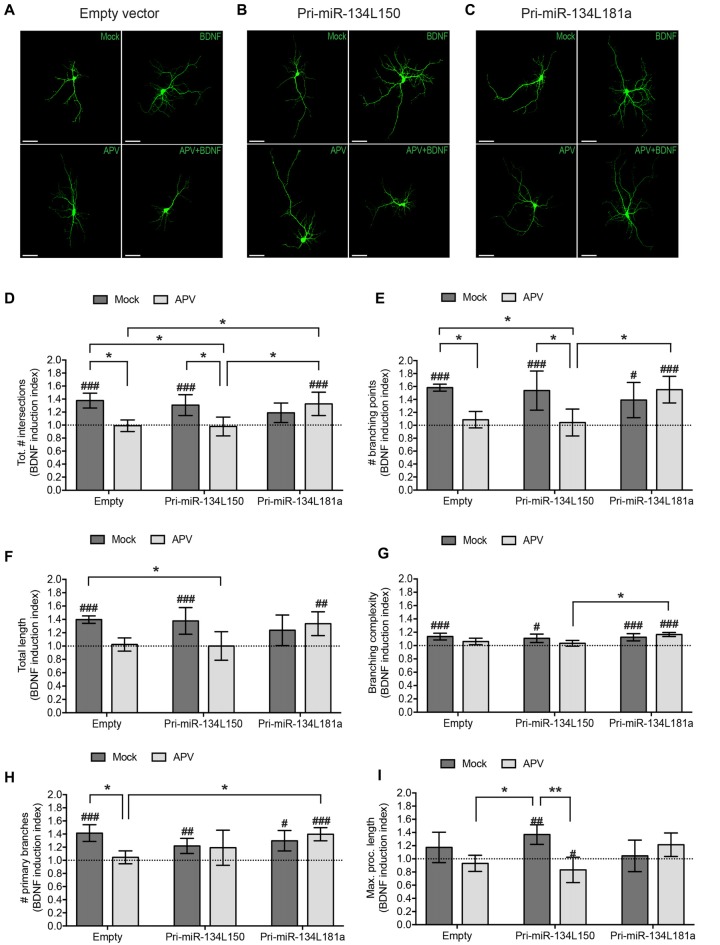
Expression of the dendritically localized pre-miR-134L181a chimera rescues BDNF-induced dendritic growth in the context of NMDAR blockade. Hippocampal neurons were transfected with the pri-miR-134L150, the pri-miR-134L181 chimeras (Figure 5E) or empty vector, subjected to drug treatment as described in Figure 4A followed by the analysis of dendritic complexity by Sholl analysis **(A–D)** or with tracking software **(E–I)**. **(A–C)** Dendritic arborization of representative GFP-transfected hippocampal neurons (DIV7) processed as indicated in Figure 4A (scale bar: 50 μm). **(D)** Sum of intersections from the Sholl profiles of the analyzed cells presented as in Figure 4. **(E-I)** Tracking software analysis of the same cells analyzed in **(A–D)**. Values represent the mean BDNF induction index for total # of intersections **(D)**, # branching points **(E)**, total length **(F)**, branching complexity **(G)**, # primary branches **(H)** or maximum length of a process **(I)** ± SD. *n* = 4 independent biological replicates. Number of analyzed cells and individual data points are indicated and analyzed in Supplementary Figure S4. Two-way ANOVA and Tukey’s tests, *P < 0.05, **P < 0.01. BDNF vs. Mock effect (see Supplementary Figure S4): Kruskal Wallis and Dunn’s test, ^#^*P* < 0.05, ^##^*P* < 0.01, ^###^*P* < 0.001.

For each data point, pictures of 8–12 developed pyramidal hippocampal neurons per experiment from three or four independent biological replicates were taken in a blinded manner on a Leica SP5 confocal microscope, using a 20× objective with a resolution of 1024 (x/y) pixels, corresponding to an image size of 459 (x/y) μm. Sholl analysis in Figure 4 was performed manually as previously described (Valluy et al., 2015), counting the intersections of dendrites with a mask made of nine concentric circles at increments of 15 μm. For experiments in Figure 6, automated Sholl analysis (Sholl profiles and sum of intersections) was performed using the “Sholl” Fiji plugin, keeping the same radius diameter. Grubb’s test was used to identify outliers. For the other indicated parameters, the same cells were processed with the WIS-Neuromath tracking software (Weizmann institute) keeping a noise level of 1. Number of analyzed cells per data point is indicated in the bar graphs of the respective experiments in Supplementary Figures S3, S4.

### Dual Luciferase Assays

Rat hippocampal (DIV5 to DIV11) and cortical neurons (DIV4 to DIV6) from three independent biological replicates were transfected respectively in technical triplicates with 200 ng or duplicates with 100 ng of Firefly and Renilla luciferase constructs. At the indicated DIV after transfection, cells were pre-incubated in APV as described before, further stimulated with BDNF for 6 h and lysed in passive lysis buffer (Promega). For pmirGLO reporter transfections, DIV5 rat cortical cells from four independent biological replicates were transfected with 500 ng pmirGLO constructs and with 500 ng pri-miR constructs in technical duplicates and 48 h later cells were lysed.

The activity of Firefly and Renilla luciferases was measured with a GloMax R96 Microplate Luminometer (Promega). Averages of Firefly/Renilla activity ratios from miR-134 reporters and mutants were further normalized with average ratios obtained from the activity of empty luciferase vectors, subjected to the same drug treatment/transfection conditions.

### Statistics

Graph bars of each data point represent means ± standard deviations of at least three independent biological replicates, except for Figures 1B,D, where boxplots were used for data presentation. Data were analyzed for statistical significance with Graphpad Prism. Normal distribution was tested with Lillefors test. Unpaired and two-tailed Student *t*-test variants were always employed. Welch’s correction was used in case of a significant variance difference between two groups. Mann-Whitney test was used as non-parametric alternative. One-way or two-way ANOVA were used in combination with *post hoc* tests for single or multiple comparisons. Kruskal-Wallis test was used as non-parametric alternative in combination with Dunn’s *post hoc* test for single or multiple comparisons. For two-way ANOVA analysis in dendritic outgrowth experiments, induction index ratios were calculated from the average sum of intersections of the biological replicates. To test the effects within the induction indices (Supplementary Figures S3, S4), cells were pooled together and Kruskall-Wallis test was used together with Dunn’s *post hoc* test. For this calculation, transfection conditions were analyzed individually.

## Results

### BDNF Treatment Induces Dendritic Enrichment of Pre-miR-134

To test our hypothesis, we treated cultured rat hippocampal neurons with BDNF and assessed pre-miR-134 localization by FISH. We applied BDNF for 2 h, since we previously found that this timescale was sufficient to induce pri-miR-134 transcription (Fiore et al., 2009). First, we performed FISH using a probe directed against the pre-miR-134 terminal loop sequence. This probe in principle is able to detect both pre- and pri-miR-134, although pre-miR-134 is likely representing the vast majority of loop-containing transcripts based on previous observations (Fiore et al., 2009). We found that 2 h BDNF stimulation significantly increased dendritic accumulation of pre-miR-134 (Figures 1A,B). This was accompanied by a significant increase in the dendritic levels of mature miR-134, as judged by FISH using a probe directed against the mature miR-134 sequence (Figures 1C,D). In contrast, BDNF did not significantly alter pre-miR-134 or miR-134 levels in the cell body (Figures 1B,D), or the combined dendritic and cell body compartment signal per area (Supplementary Figures S1A,B). We were further able to confirm our results from FISH using an independent approach based on a previously described compartmentalized neuron culture system (Bicker et al., 2013). BDNF treatment of the process-enriched compartment promoted an enrichment of pre-miR134 and miR-134 in neuronal processes compared to the untreated condition (Supplementary Figures S1C,D). In contrast, treatment of the cell body compartment showed pre-miR-134 enrichment in the cell body compartment, which is consistent with a rapid transcriptional induction of pri-miR-134 in the nucleus. The FISH probe used in Figure 1A does not distinguish between pre- and pri-miR-134, since it is directed against the terminal loop region. In order to estimate the contribution of pri-miR-134 more specifically, we performed smFISH using an 836 nt long probe spanning three exons of the Mirg transcript including the miR-134 containing exon (Exon134_485; Figure 1E, Supplementary Figure S1E). Thereby, we could detect a specific signal that was induced by BDNF treatment, consistent with BDNF-dependent upregulation of pri-miR-134 (Fiore et al., 2009). Importantly, the Exon134_485 signal was restricted to the nucleus, whereas abundant dendritic staining was observed when using a probe against the known dendritic mRNA CamK2α. Conversely, no nuclear staining was detected when using a probe against the unrelated bacterial gene DapB (Figures 1E,F, Supplementary Figure S1E). This result strongly suggests that the majority of dendritic signal observed in Figures 1A–D is derived from pre-miR-134. Taken together, dendritic localization of pre-miR-134 is increased by BDNF.

### Dendritic Accumulation of Pre-miR-134 Is NMDA Receptor-Dependent

As a next step, we investigated the signaling pathways that are involved in BDNF-dependent dendritic pre-miR-134 accumulation. Multiple functions of BDNF, including its positive effect on dendritogenesis, were shown to depend on functional NMDA receptors (NMDARs; Park and Poo, 2013). We therefore interrogated an involvement of NMDAR-dependent signaling using the selective NMDAR antagonist APV. Neurons were pre-treated for 30 min with APV before BDNF application for 2 h. Using qPCR analyses of our compartmentalized neuron culture system, we found that, consistent with our results from Figures 1A,B and Supplementary Figure S1C, BDNF treatment of the process compartment led to a significant enrichment of pre-miR-134 in neuronal processes compared to the cell body (Figure 2A). BDNF-dependent process enrichment of pre-miR-134 was strongly attenuated by APV, demonstrating that BDNF mediates its effect via NMDAR activation. Conversely, none of the treatments significantly altered pre-miR-134 enrichment when applied to the cell body (Figure 2B), suggesting a selective engagement of dendritic signaling mechanisms. A long Pum2 mRNA isoform whose localization is not affected by BDNF (Fiore et al., 2009) served as a negative control (Supplementary Figures S2A,B). In principle, potential effects of BDNF/APV on miRNA transcription and/or processing could obscure the results obtained for dendritic accumulation of pre-miR-134. Therefore, we first assessed the effect of APV on BDNF-dependent miR-134 transcription. Using qPCR with RNA obtained from whole-cell neuronal lysates, we found that APV had no effect on the observed neuron-wide increase in pri-miR-134 and mature miR-134 upon BDNF treatment (Figure 2C). c-Fos and Dhx36 mRNA were used as positive and negative control for BDNF, respectively. The functionality of APV was validated by measuring glutamate-mediated cFos induction (Supplementary Figure S2C). To unequivocally assess the effect of APV on pre-miR-134 localization without contribution of transcriptional effects, we decided to monitor the subcellular localization of synthetic, fluorescently labeled pre-miR-134, which bypasses endogenous transcription and pri-miRNA processing. Towards this aim, *in vitro* synthesized, Cy3-labeled pre-miR-134 was transfected into hippocampal neurons which were concurrently treated with BDNF in the presence or absence of APV. Consistent with our results obtained with compartmentalized cultures (Figure 2A), APV pre-treatment reduced the percentage of synthetic pre-miR-134 granules present in dendrites (Figures 2D,E). Importantly, the total number of transfected granules per cell was comparable between the conditions, suggesting that APV had no effect on pre-miR-134 processing or stability (Figure 2F). In conclusion, our data suggest a critical involvement of NMDAR-dependent signaling in dendritic pre-miR-134 accumulation.

### miR-134 Dendritic Target Pum2 Is Not Repressed by BDNF in Conditions of NMDAR Blockade

Next, we investigated whether NMDAR-dependent dendritic pre-miR-134 accumulation was involved in the regulation of dendritic miR-134 target mRNAs. Therefore, we tested the effect of APV treatment on the translation of a destabilized luciferase reporter under the control of the long isoform of the Pum2 3′UTR (Pum2-3′UTR-long). We recently showed that Pum2-3′UTR-long containing transcripts are dendritically localized and translationally repressed by miR-134 in a DHX36-dependent manner (Bicker et al., 2013; Fiore et al., 2014). In accordance with our previous data obtained with KCl treatment (Fiore et al., 2009), BDNF treatment significantly repressed Pum2-3′UTR-long reporter gene activity in both primary hippocampal and cortical neurons (Figures 3A,B). BDNF-dependent repression of Pum2 was completely abolished by APV pre-treatment, suggesting that this effect required functional NMDAR. Notably, the de-repressive effect of APV required miR-134, since activity of a Pum2 reporter containing a mutated miR-134 binding site (Pum2-3′UTR-long-Mut134) was not further elevated by APV. Similar results were observed in both hippocampal and cortical neurons (Figures 3A,B). These data suggest that NMDAR-dependent pre-miR-134 accumulation in neuronal dendrites is involved in the repression of endogenous miR-134 target mRNAs.

### miR-134-Mediated BDNF-Induced Dendritic Outgrowth Is Blocked by Paradigms Interfering With Dendritic Pre-miR-134 Accumulation

In the next set of experiments, we addressed the functional relevance of NMDAR-dependent pre-miR-134 accumulation in neuronal dendrites. Therefore, we focused on BDNF-induced dendritic outgrowth, since it requires both NMDAR activity and miR-134-dependent repression of Pum2 (McAllister et al., 1996; Fiore et al., 2009). Dendritogenesis in immature neurons (DIV7, see Figure 4A for description of experimental manipulations) was assessed by confocal microscopy followed by Sholl analysis. First, we validated that our protocol using a 3 h bath application of BDNF led to a robust increase in dendritic complexity compared to control conditions (Figures 4B,C, Supplementary Figure S3A) as judged by the average total number of intersections summed from the Sholl profiles of the analyzed cells. Next, we assessed the effect of shRNA-mediated knockdown of DHX36, which we previously showed to be required for pre-miR-134 dendritic accumulation (Bicker et al., 2013). Knockdown of DHX36 by two independent shRNAs completely abolished BDNF-induced dendritic outgrowth, whereas transfection of an unrelated control shRNA had no effect (Figures 4D,E, Supplementary Figures S3B,C). Similarly, APV but not TTX treatment prior to BDNF stimulation completely prevented the dendrite growth-promoting effect of BDNF on hippocampal neurons (Figures 4F,G, Supplementary Figures S3D,E). Together, these two lines of evidence are consistent with a requirement of pre-miR-134 dendritic accumulation for BDNF-induced dendrite outgrowth. Next, we addressed a causal involvement of miR-134 in NMDAR-dependent BDNF-induced dendritogenesis. We reasoned that if reduced dendritic miR-134 levels underlie APV-mediated inhibition of dendrite outgrowth, we should be able to rescue this phenotype by restoring dendritic miR-134 levels. To this aim, we attempted to elevate dendritic miR-134 levels by transfecting synthetic miR-134 duplex RNA before drug stimulations (Figure 4H, Supplementary Figures S3F–H). As previously reported (Fiore et al., 2009), miR-134 duplex RNA transfection reduced BDNF-induced dendritic outgrowth, thereby confirming that overexpression of functional miR-134 was achieved (Figure 4H, Mock bars). In addition, in the presence of APV, dendrites from miR-134 transfected neurons were significantly more complex compared to those transfected with control duplex, suggesting that elevating miR-134 levels abrogated the inhibitory function of APV (Figure 4H, APV bars, Supplementary Figures S3F–H). Together, these data suggest that elevation of dendritic miR-134 levels is an important downstream mediator of NMDAR-dependent dendritic outgrowth.

### Design and Validation of a Dendritically Localized Pre-miR-134L181a Chimera

Although our results so far provided evidence for an involvement of miR-134 in BDNF- and NMDAR-dependent dendritogenesis, they did not conclusively demonstrate a causal role of dendritically transported pre-miR-134 in this process. To address this issue, we decided to use a pre-miR-134 chimera that bypasses the APV-induced pre-miR-134 transport block for rescue experiments. Therefore, we substituted the pre-miR-134 loop region, which is necessary for DHX36-dependent pre-miR-134 dendritic localization (Bicker et al., 2013) and therefore likely responsible for the APV sensitivity, with the loop of another dendritically localized pre-miRNA (pre-miR-181a, Sambandan et al., 2017). As a negative control, we used the cell body-retained pre-miR-134L150 chimera (Bicker et al., 2013; Figure 5A). To assess the localization of these chimeras, we *in vitro* synthesized Cy3-labeled pre-miRNAs, transfected the RNAs in DIV7 hippocampal neurons and fixed cells after 3 h (Figure 5B). As expected, pre-miR-134L150 chimeras showed a lower percentage of dendritically localized granules compared to the wild-type (wt) pre-miR-134 construct. On the other hand, dendritic localization of the pre-miR-134L181a chimera was comparable to wt pre-miR-134, thereby confirming the suitability of this tool for our further studies (Figure 5C). The total number of pre-miRNA containing granules per cell was comparable between the different sequences, suggesting that loop substitutions within pre-miRNAs had no adverse effects on their stability (Figure 5D). Furthermore, the expression of a luciferase vector under the control of 3× miR-134 binding sites was significantly repressed by the co-transfection of Pol II-dependent expression vectors harboring pre-miR-134 chimeric sequences within the context of the endogenous pri-miR-134 flanking sequence (Figure 5E). In conclusion, we could validate the use of the pre-miR-181a loop as a dendritic targeting element for pre-miR-134.

### A Dendritically Localized Pre-miR-134L181a Chimera Rescues BDNF-Induced Dendritic Outgrowth in the Presence of APV

We then proceeded with the transfection of the pri-miR-134 chimeras used in Figure 5E and analyzed their effect on BDNF-induced dendritic outgrowth in the presence of APV. As expected based on our previous results, APV treatment was able to abolish the dendrite growth-promoting effect of BDNF when either an empty vector or the cell body-retained pri-miR-134L150 chimera were transfected (Figures 6A,B,D, Supplementary Figures S4A,B,D). In contrast, transfection of the dendritically localized pri-miR-134L181a chimera rescued BDNF-induced dendritic outgrowth in the presence of APV (Figures 6C,D, Supplementary Figures S4C,D), thereby mimicking the results of the miR-134 duplex transfection (Figure 4H). Given the importance of this experiment for our working hypothesis, we decided to perform a more in-depth analysis of dendritic parameters using the WIS-Neuromath tracking software (Rishal et al., 2013). In accordance with our Sholl analysis and previous reports (McAllister et al., 1996), BDNF induced an increase in the number of branching points, total process length and primary dendrite sprouting, which was blocked by APV treatment in the context of empty vector or pri-miR-134L150 chimera transfection. Conversely, the induction was preserved upon pri-miR-134L181a transfection (Figures 6E–H Supplementary Figures S4E–H). Moreover, the branching complexity of the cells, represented by the ratio of branching points number to total length, showed a similar trend (Figure 6G, Supplementary Figure S4G). Surprisingly, we observed both a BDNF- and an APV-dependent increase in maximum process length specifically in the context of pri-miR-134L150 transfection (Figure 6I, Supplementary Figure S4I). In line with previous observations (McAllister et al., 1996), this finding may suggest two different mechanisms of action for BDNF and APV and an additional function for cell body-retained pre-miR-134 in the control of process length. A number of other parameters did not show any consistent changes between the different conditions (Supplementary Figures S4J–L). In conclusion, our results demonstrate that the dendrite growth-inhibitory effect of NMDAR blockade is mediated by decreased pre-miR-134 levels specifically in dendrites.

## Discussion

In this study, we show that local BDNF treatment of neuronal processes induces pre-miR-134 dendritic enrichment via an NMDAR-dependent pathway. Furthermore, we found that this pathway was required for activity-dependent dendritogenesis. Therefore, our work identifies pre-miRNA dendritic localization as a novel mechanism involved in the regulation of activity-dependent neuronal development.

Multiple lines of evidence already suggested that neuronal activity or RBPs exert a tight regulation over every step of miRNA biogenesis (Aksoy-Aksel et al., 2014; Sambandan et al., 2017). For example, pri-miR-134 transcription and mature miR-134 levels are increased upon different activity stimuli (BDNF, KCl, Picrotoxin) and during *in vitro* development of neuronal cultures (Schratt et al., 2006; Fiore et al., 2009, 2014). On the other hand, relatively little is known about mechanisms regulating pre-miRNA subcellular localization in neurons. In contrast, BDNF was previously implicated in the control of neuronal pre-miRNA processing. For example, BDNF increases levels of Dicer and the RBP Lin28 to downregulate pre-let-7 biogenesis and mediate dendritic outgrowth (Huang et al., 2012). More recently, we showed that BDNF stimulation induces the dissociation of TRBP from endoplasmic reticulum-localized Dicer, thereby reducing pre-miR-16 processing and promoting dendritic growth (Antoniou et al., 2018). In the present study, we did not obtain direct evidence for BDNF- or APV-dependent regulation of miR-134 biogenesis downstream of transcription (Figure 2C). Moreover, restoring dendritic pre-miR-134 levels was sufficient to rescue deficits in activity-dependent dendritogenesis inflicted by NMDAR inhibition (Figure 6). Therefore, our results are most consistent with a model whereby BDNF stimulates pre-miR-134 transport to dendrites in an NMDAR-dependent manner, followed by BDNF/NMDAR-independent local processing of pre-miR-134 in dendrites.

In contrast to miRNAs, dendritic transport of mRNAs has been extensively studied, which helped to build a relatively precise model of dendritic mRNA transport. In this model, specific sequence motifs within dendritically localized mRNAs are bound co-transcriptionally by RBPs, which, when in the cytoplasm, are assembled into larger ribonucleoprotein particles (RNPs). RNPs are further re-directed to a dendritic transport machinery, where the mRNA is kept in a translationally silenced state until the final destination in dendrites is reached (Besse and Ephrussi, 2008). Notably, neurotrophins and NMDAR-dependent synaptic activity promote the dendritic transport of specific mRNA transcripts, such as β-Actin, Arc and CamK2α, which are subsequently translated in response to synaptic activation (Bramham and Wells, 2007; Yoon et al., 2016). Combining our work with those findings, it is conceivable that the life of a dendritically localized pre-miRNA undergoes a very similar path. For example, pre-miRNAs that are destined to dendrites could be bound and incorporated in larger RNPs and thereby escape from Dicer-mediated processing until reaching their dendritic destination. This view is supported by our recent findings that pre-miR-134 is bound by DHX36, an RBP that is a component of transport RNPs and Dicer/Ago-containing protein complexes (Hock et al., 2007; Fritzsche et al., 2013). Furthermore, DHX36 counteracts Dicer-dependent cleavage of pre-miR-134 *in vitro* (Bicker et al., 2013).

At the functional level, we found that DHX36 knockdown phenocopies the effects of APV treatment both with regard to dendritogenesis and pre-miR-134 transport (Figures 4D–G, Bicker et al., 2013). Therefore, it is tempting to speculate that blockade of dendritic NMDARs could disrupt the direct association of pre-miR-134 and DHX36 or, indirectly, of the pre-miR-134/DHX36 complex with the transport machinery. Mechanistically, we envision that reduced calcium influx upon NMDAR blockade could alter post-translational modifications of DHX36 or the kinetics of calcium-dependent protein-protein or protein-RNA interactions, thereby modulating pre-miR-134 transport efficiency (see for example Muslimov et al., 2014 and Ichinose et al., 2015). On the other hand, NMDAR blockade leads to a general reduction of mRNA dendritic localization (Bramham and Wells, 2007; Yoon et al., 2016), suggesting an involvement of general activity-regulated RNP assembly processes.

Concerning the spatial regulation of pre-miRNA transport, our results support a model whereby BDNF stimulates dendritic pre-miR-134 accumulation via the local stimulation of dendritic TrkB receptors (Figure 2A). Since activation of TrkB receptors in cell bodies did not promote pre-miR-134 dendritic enrichment, we consider it unlikely that activation of signaling pathways in the cell body significantly contributes to the dendritic pre-miR-134 pool (Figure 2B). However, our experimental setup does not completely rule out a contribution of transcriptional activation to increased dendritic pre-miR-134 levels following BDNF stimulation.

Our results are further consistent with a wealth of previous data that document the relevance of NMDAR-dependent signaling in activity-dependent dendritogenesis. Similar to BDNF secretion, NMDAR stimulation promotes dendritic branching in *Xenopus* tectal neurons and in the rat supraoptic nucleus, whereas application of NMDAR antagonists triggers a decrease in dendritic complexity (Rajan and Cline, 1998; Chevaleyre et al., 2002). Depending on age and context, though, APV stimulation may also lead to an increase in dendritic branching and growth (McAllister, 2000). Consistently, in our Sholl analyses APV induces dendritic complexity both at basal levels and upon transfection of the cell body-retained pre-miR-134L150 chimera (Supplementary Figure S4D).

There is also ample evidence for a functional crosstalk between BDNF- and NMDAR-dependent signaling. For example, in developing hippocampal neurons, BDNF application elicits dendritic calcium transients which depend on TrkB signaling, voltage-gated sodium and calcium channels (Lang et al., 2007). Moreover, combined activation of the two pathways results in sustained dendritic branching and stabilization, and also in autocrine dendritic BDNF focal secretion, which exerts paracrine pro-growth effects in dendrites located in close vicinity (Park and Poo, 2013). BDNF-induced dendritic outgrowth requires NMDAR activity, as shown in layer 4 pyramidal neurons from ferret cortical brain slices (McAllister et al., 1996). Importantly, in our short-timed stimulation protocol, preventive blockade of NMDARs, but not of voltage-gated sodium channels (VGSCs), reduces BDNF-induced outgrowth (Figures 4F,G). Conversely, longer treatments with TTX, as well as with APV, CNQX (an AMPA receptor blocker) and Nifedipine (an L-type Ca^++^ channel blocker), were shown to impair dendritic induction (McAllister et al., 1996). These results suggest that VGSCs might contribute to BDNF-induced dendritic growth with slower kinetics than NMDARs. On the other hand, our data (Figures 3, 4H, 6) indicate that a short-term NMDAR blockade is sufficient to deregulate BDNF-induced and miR-134-dependent dendritic protein synthesis in developing neurons.

With regard to miRNA target regulation, our data point towards a failure in BDNF-induced miR-134-mediated Pum2 repression (Figure 3). Pum2 is a dendritically localized RBP that negatively regulates the translation of dendritically localized mRNAs, e.g., those encoding the translation factor eIF-4E or VGSCs (Vessey et al., 2010; Driscoll et al., 2013). We therefore speculate that under NMDAR blockade, Pum2 de-repression caused by reduced dendritic miR-134 might lead to reduced protein synthesis and sodium channel expression, thereby impairing dendritic outgrowth.

Many neurodevelopmental and neurodegenerative disorders are characterized by dendritic atrophy, suggesting defects in activity-dependent dendritogenesis (Kulkarni and Firestein, 2012; Martínez-Cerdeño, 2017). Since BDNF and NMDAR signaling have been implicated in these disorders, it would be informative to investigate whether pre-miR-134 dendritic localization is affected in pyramidal neurons from patients or from animal disease models. If this turns out to be the case, restoring dendritic miR-134 levels, e.g., by similar strategies as presented in this study, could prove a valuable strategy to restore the neuromorphological defects associated with these devastating disorders.

## Author Contributions

FZ designed the study, performed most of the experiments and wrote the manuscript. SB performed *in situ* hybridizations, experiments with compartmentalized cultures and supervised the project. GS wrote the manuscript and supervised the project.

## Conflict of Interest Statement

The authors declare that the research was conducted in the absence of any commercial or financial relationships that could be construed as a potential conflict of interest.
