# Supplementary material for: Activity-Dependent Pre-miR-134 Dendritic Localization Is Required for Hippocampal Neuron Dendritogenesis
Source: Front Mol Neurosci. 2018 Jun 11;11:171. doi: 10.3389/fnmol.2018.00171 (PMC6004952; doi:10.3389/fnmol.2018.00171)
Supplement: Supplementary file 1 [file Data_Sheet_1.pdf]

Supplementary Material

Activity-dependent pre-miR-134 dendritic localization is required for hippocampal neuron dendritogenesis

Zampa, F., Bicker, S. *, Schratt, G.*

*** Correspondence:**

Gerhard Schratt, Gerhard.schratt@hest.ethz.ch

Silvia Bicker, silvia.bicker@hest.ethz.ch

Primers and probes

RT-qPCR

Pre-miR-134_fw: TGTGACTGGTTGACCAGAGGG

Pre-miR-134_rev: GGTGACTAGGTGGCCCACAG

Pre-miR-7a-2_fw: CTGTCTGGAAGACTAGTGATTTTGTTG

Pre-miR-7a-2_rev: GACTTGTTGTTGGACACAGACACA

GAPDH_fw: GCCTTCTCTTGTGACAAAGTGGA

GAPDH_rev: CCGTGGGTAGAGTCATACTGGAA

c-fos_fw: CATCATCTAGGCCCAGTGGC

c-fos_rev: AGGAACCAGACAGGTCCACATCT

Pri-miR-134_fw: GGCCCCCGGTATCACCTTAG (combined with pre-miR-134_rev)

Dhx36_fw: GCATCCGGCCCACCTTAA

Dhx36_rev: CTCTTCTCGCCGTTCATCCA

U6_fw: CTCGCTTCGGCAGCACA

U6_rev: AACGCTTCACGAATTTGCGT

Pre-miRNA *in vitro* transcription templates

Pre-miR-134_fw1: AATTTAATACGACTCACTATAGGTGACTGGTTGACCAGAG

Pre-miR-134_rev1: GGTCAACCAGTCACCTATAGTGAGTCGTATTAAATT

Pre-miR-134_fw2: GGGCGTGCACTTTGTTCACCCTGTGGGCCACCTAGTCACCAA

Pre-miR-134_rev2:

TTGGTGACTAGGTGGCCCACAGGGTGAACAAAGTGCACGCCCCTCT

Pre-miR-134L150_fw2: GGGCTGTGCCTCAGACCCTGTGGGCCACCTAGTCACCAA

Pre-miR-134L150_rev2:

TTGGTGACTAGGTGGCCCACAGGGTCTGAGGCACAGCCCCTCT

Pre-miR-134L181a1_fw2:

GGGTTGGAATTCAAATAAAACTGTGGGCCACCTAGTCACCAA

Pre-miR-134L181a1_rev2:

TTGGTGACTAGGTGGCCCACAGTTTTATTTGAATTCCAACCCCTCT

Pri-miRNA plasmids

Pri-miR-134-HindIII_fw: CAGTAAGCTTGAGCAGCTGATCCATCTGTAGGC

Pri-miR-134-XhoI_rev: CAGTCTCGAGCTCATGGCATCTTATGTAAACATACACC

Pri-miR-134L150_fw: GCTGTGCCTCAGACCCTGTGGGCCACCTAGTCACCAAC

Pri-miR-134L150_rev: TCGACCTGTGTGGAATGTGGG

Pri-miR-134L181a1_fw: TGGAATTCAAATAAAACTGTGGGCCACCTAGTC

Pri-miR-134L181a1_rev: TATTTGAATTCCAACCCCTCTGGTCAACCAGTC

3x miR-134pbs insert for cloning:

TCTAGACCCCTCTGGTCAACCAGTCACACTCCCCTCTGGTCAACCAGTCACACTCC
CCTCTGGTCAACCAGTCACAGCTA

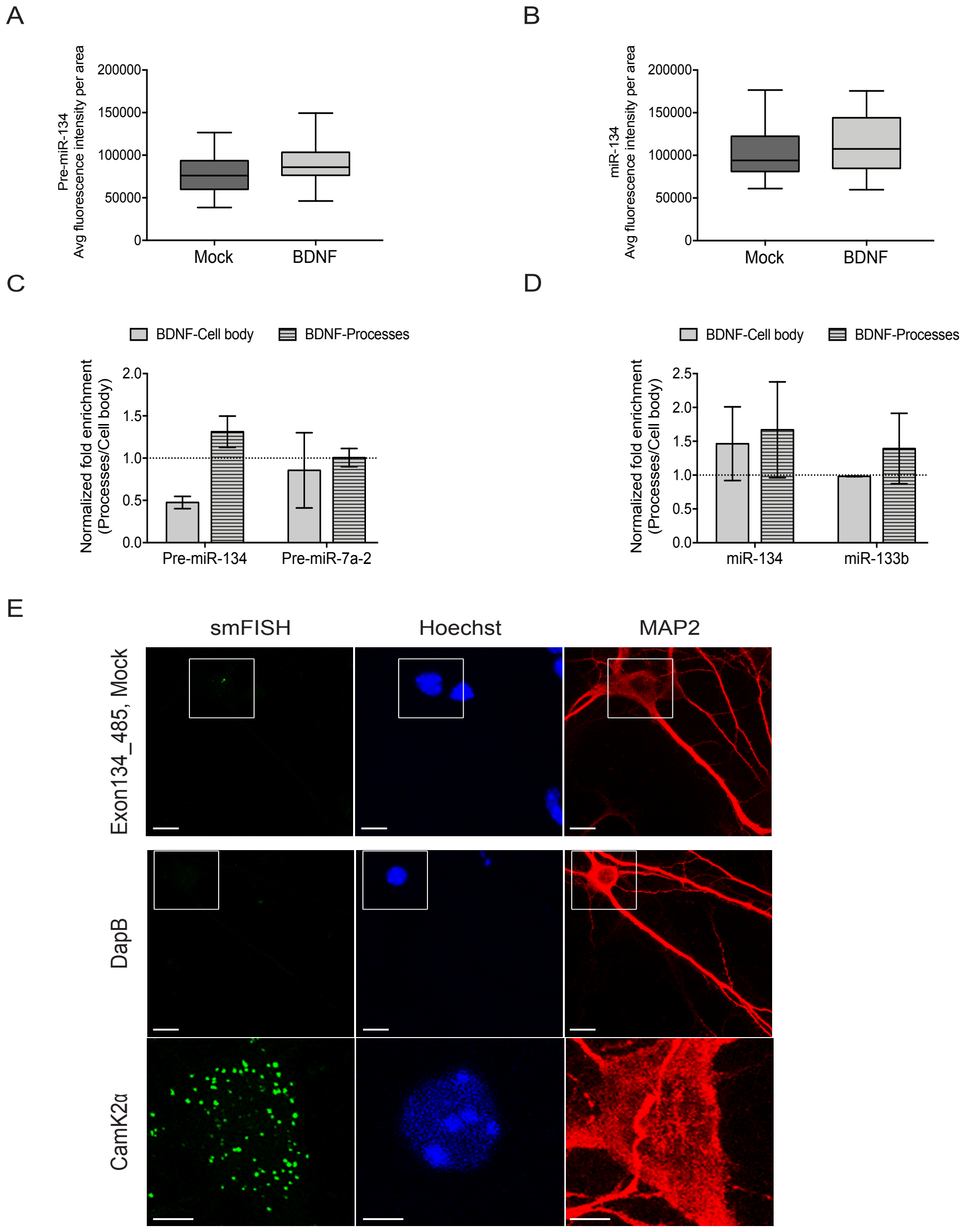

Supplementary Figure S1

(A, B) Quantification of pre-miR-134 or miR-134 total signal per area. **(C, D)** qPCR using RNA extracted from both the process and cell body compartment of developed hippocampal neurons cultured on filter inserts and treated for 2h with BDNF selectively in the process or cell body compartment. Values represent the mean of the relative process enrichment (ratio process vs. cell body expression) of pre-miR-134 **(C)** and miR-134 **(D)** ± S.D. Values obtained for mock-treated neurons are set to one. n=2 independent biological experiments. Pre-miR-7a-2 and miR-133b were used as negative control. **(E)** Completion of the set of the representative pictures in Fig. 1 E, F. Exon134_485 (Mock) and DapB (scale bar: 10μm), CamK2α (scale bar: 5μm).

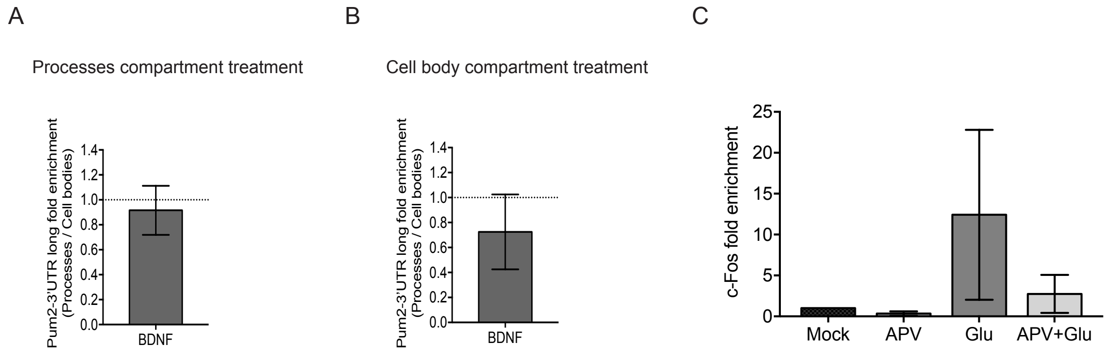

Supplementary Figure S2

(A, B) Pum2 3'UTR RNA levels do not change upon BDNF stimulation in dendritic and cell body compartments. **(C)** APV blocks glutamate-induced increase in c-Fos transcription. Values represent c-Fos fold enrichement ± S.D. n=4 independent biological experiments.

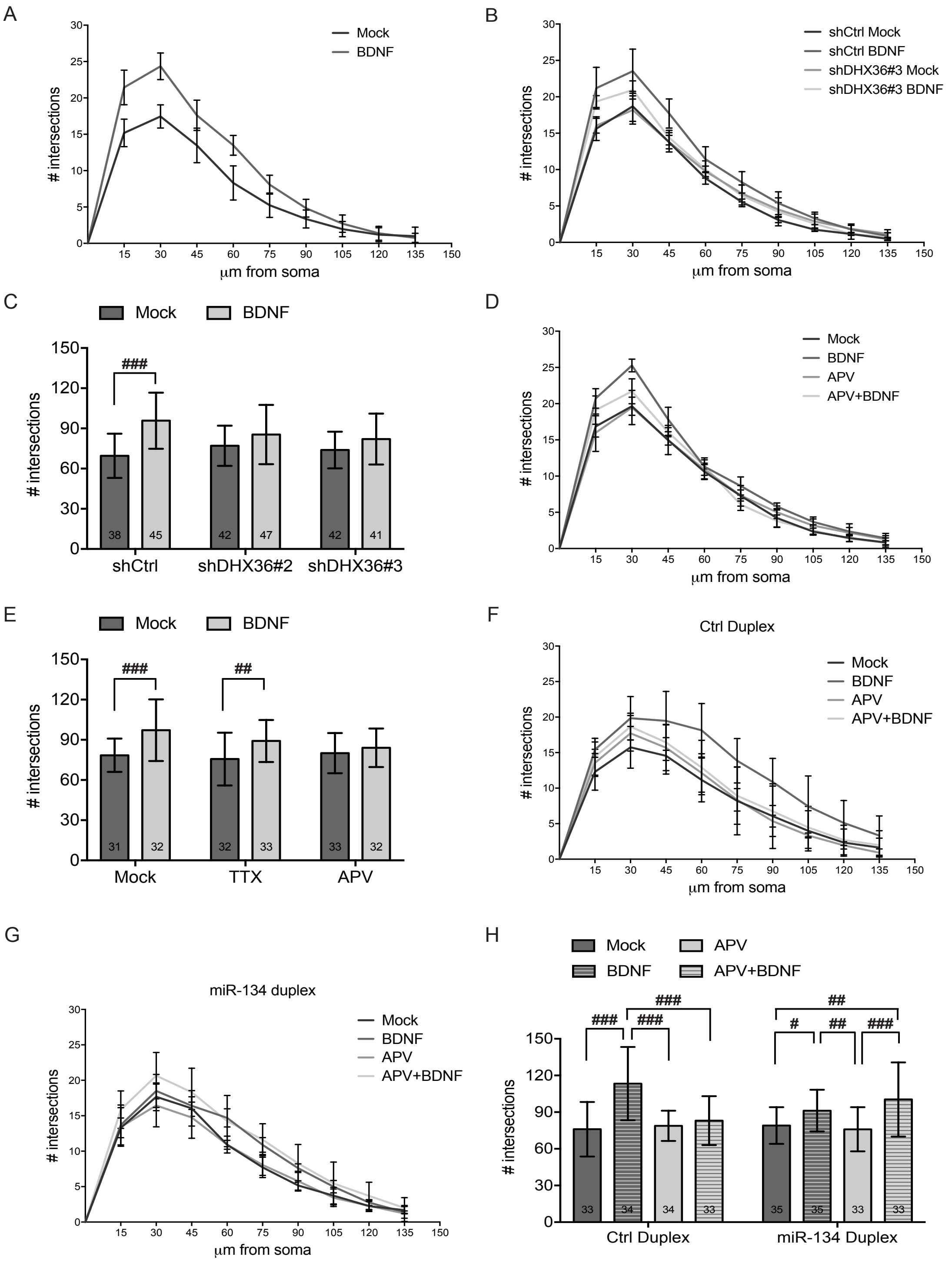

Supplementary Figure S3

(A) Mean of the Sholl profile averages from biological replicates of Fig. 4B, C. **(B)** Mean of the Sholl profiles averages from biological replicates and **(C)** mean number of intersections for raw conditions presented as induction indeces in Fig. 4D, E, in Fig. 4F, G **(D, E)** and in Fig.4 H **(F-H)**. Values indicate the number of cells analyzed per condition. (C, E) One-way ANOVA and Bonferroni test for single Mock vs BDNF comparison. (H) Kruskal-Wallis test and Dunn's test for multiple comparisons within transfection conditions (ctrl duplex and miR-134 duplex), $^{\#}P < 0.05$, $^{\#\#}P < 0.01$, $^{\#\#\#}P < 0.001$.

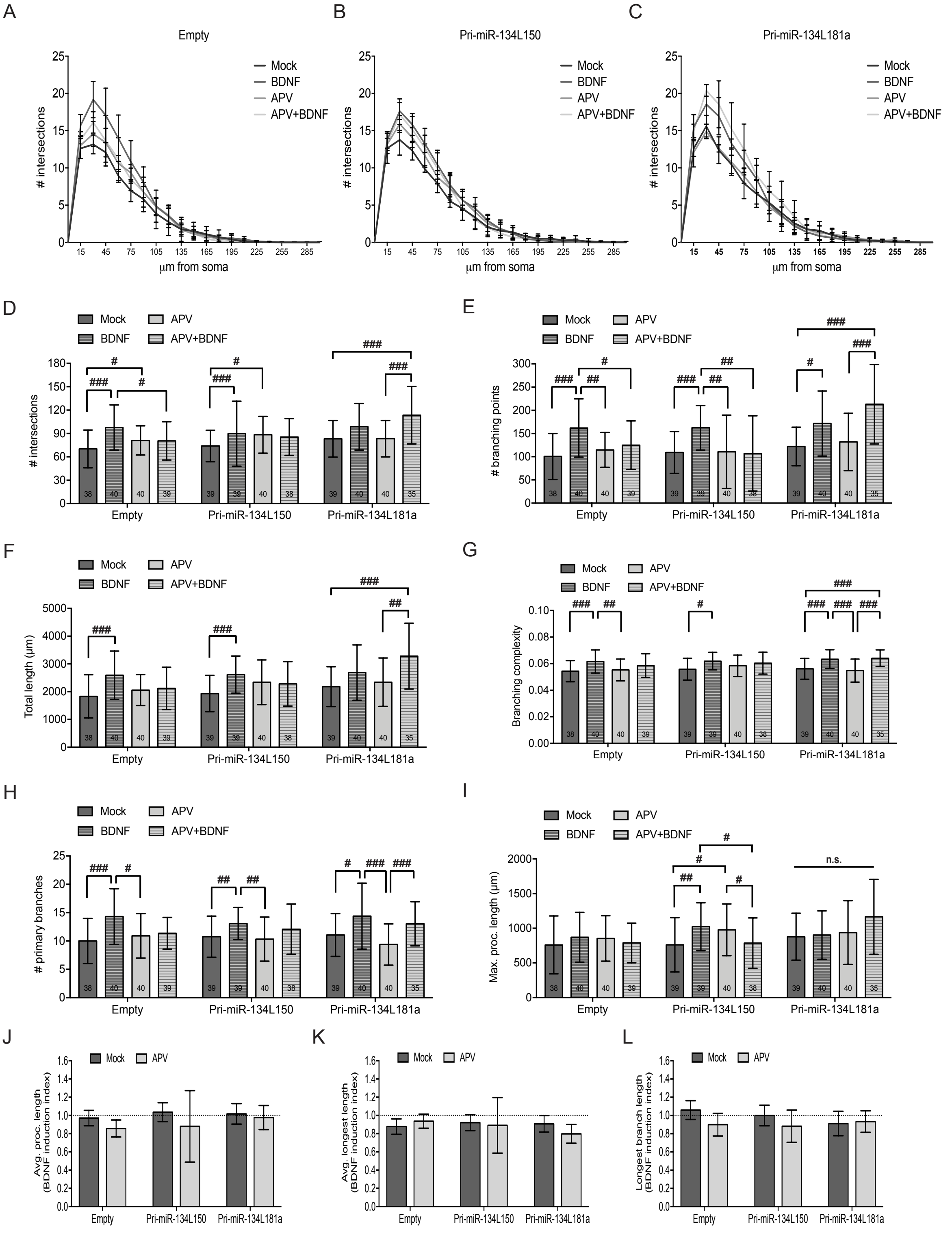

Supplementary Figure S4

(A-C) Mean of the Sholl profile averages from biological replicates of Fig. 6 A-B **(D-I)** Mean number of intersections for raw conditions presented as induction indeces in Fig.6. Values indicate the number of cells analyzed per condition. Kruskal-Wallis test and Dunn's test for multiple comparisons within transfection conditions (empty, pri-miR-134L150, pri-miR-134L181a), $^{\#}$ $P < 0.05$, $^{\#\#}$ $P < 0.01$, $^{\#\#\#}$ $P < 0.001$. **(J-L)** BDNF induction indeces from cell length-related parameters show no change. **(J)** Average process length. **(K)** Average length of the longest process. **(L)** Longest branch length. No significant interactions were detected from analysis of single conditions.
